# Treatment outcomes for patients with Middle Eastern Respiratory Syndrome Coronavirus (MERS CoV) infection at a coronavirus referral center in the Kingdom of Saudi Arabia

**DOI:** 10.1186/s12879-016-1492-4

**Published:** 2016-04-21

**Authors:** Mohammed Al Ghamdi, Khalid M. Alghamdi, Yasmeen Ghandoora, Ameera Alzahrani, Fatmah Salah, Abdulmoatani Alsulami, Mayada F. Bawayan, Dhananjay Vaidya, Trish M. Perl, Geeta Sood

**Affiliations:** King Fahd General Hospital, Jeddah, Kingdom of Saudi Arabia; Department of Medicine, Division of General Internal Medicine, The Johns Hopkins University, School of Medicine, Baltimore, Maryland USA; Department of Internal Medicine, Division of Infectious Diseases, The Johns Hopkins University, School of Medicine, Baltimore, 21224 Maryland USA

**Keywords:** Middle Eastern Respiratory Syndrome coronavirus, MERS CoV, Coronavirus, Survival, Treatment outcome

## Abstract

**Background:**

Middle Eastern Respiratory Syndrome coronavirus (MERS-CoV) is a poorly understood disease with no known treatments. We describe the clinical features and treatment outcomes of patients with laboratory confirmed MERS-CoV at a regional referral center in the Kingdom of Saudi Arabia.

**Methods:**

In 2014, a retrospective chart review was performed on patients with a laboratory confirmed diagnosis of MERS-CoV to determine clinical and treatment characteristics associated with death. Confounding was evaluated and a multivariate logistic regression was performed to assess the independent effect of treatments administered.

**Results:**

Fifty-one patients had an overall mortality of 37 %. Most patients were male (78 %) with a mean age of 54 years. Almost a quarter of the patients were healthcare workers (23.5 %) and 41 % had a known exposure to another person with MERS-CoV. Survival was associated with male gender, working as a healthcare worker, history of hypertension, vomiting on admission, elevated respiratory rate, abnormal lung exam, elevated alanine transaminase (ALT), clearance of MERS-CoV on repeat PCR polymerase chain reaction (PCR) testing, and mycophenolate mofetil treatment. Survival was reduced in the presence of coronary artery disease, hypotension, hypoxemia, CXR (chest X-ray) abnormalities, leukocytosis, creatinine >1 · 5 mg/dL, thrombocytopenia, anemia, and renal failure. In a multivariate analysis of treatments administered, severity of illness was the greatest predictor of reduced survival.

**Conclusions:**

Care for patients with MERS-CoV remains a challenge. In this retrospective cohort, interferon beta and mycophenolate mofetil treatment were predictors of increased survival in the univariate analysis. Severity of illness was the greatest predictor of reduced survival in the multivariate analysis. Larger randomized trials are needed to better evaluate the efficacy of these treatment regimens for MERS-CoV.

## Background

Coronaviruses cause a spectrum of illness from asymptomatic disease to respiratory failure. Early reports of coronavirus infections suggested that most infections were mild until the 2003 SARS epidemic that was associated with significant morbidity and mortality [1]. In September 2012, a novel coronavirus was identified in a 60-year old man in Saudi Arabia [2]. A second case was identified in a Qatari patient hospitalized in the United Kingdom [3]. The two coronaviruses were genetically identical and similar to isolates obtained from bats [4]. In July 2013, the coronavirus study group named this new virus Middle East respiratory syndrome coronavirus (MERS–CoV) [5].

As of December 21, 2015, there have been 1625 cases worldwide with 586 deaths [6]. The epidemiology and clinical manifestations of this disease have described a spectrum of illness from asymptomatic infection to severe respiratory failure and death. The overall mortality rate remains at 37 % [7–15]. Importantly, there are no known effective treatments. In 2014 there was an increase in MERS-CoV cases reported from the Jeddah region of Saudi Arabia. To describe the changing epidemiology and outcomes, we report the clinical features and treatment outcomes of patients admitted to a regional referral hospital in Jeddah, Saudi Arabia.

## Methods

### Study setting and participants

King Fahd General Hospital is an 800-bed hospital in Jeddah, Kingdom of Saudi Arabia and is a regional coronavirus referral center. There are 36 ICU beds and one Infectious Disease physician that serves the hospital. Between January through December 2014, all patients admitted or transferred to King Fahd Hospital with a positive MERS coronavirus PCR from clinical nasal swabs or nasopharyngeal aspirates were included.

### Molecular methods

All PCR testing was performed at the Ministry of Health Regional Lab in Jeddah. The MagNa Pure Compact/MagNa Pure 96 (Roche) automated system was used to extract RNA from samples. Primers and probes for upE and Orf 1a targets of MERS-CoV were used from TIB MOLBIOL (Germany) along with Master Mix from Roche for the Light Cycler 480 II (Roche) were used to amplify upE and Orf 1a gene targets. Samples that tested positive for both upE and Orf 1a gene targets with a cycle threshold time of less than 37 were considered confirmed cases. Positive and negative controls were used to monitor the amplification process & to check for any inhibition of amplification.

### Case review and definitions

Medical charts for all patients were reviewed and data abstracted on standardized data collection forms by an infectious disease trained physician. Demographic, clinical and laboratory data were entered into a database. To understand the epidemiology, age was categorized as <30, 30–60 and >60. Hypotension was defined as blood pressure <90/60 mm Hg, tachypnea as a respiratory rate greater than 16, hypoxia as an oxygen saturation <90 %, thrombocytopenia as platelets <150,000/cubic millimeter, leukopenia was defined as a white blood cell count <5000 cells/cubic millimeter and leukocytosis as a white blood cell count >10,000 cells/cubic millimeter. Renal insufficiency was defined as a creatinine >1.5 mg/dL. Liver function abnormalities were defined as a lactate dehydrogenase (LDH) >300 U/liter, alanine transaminase (ALT) > 50 U/Liter and aspartate aminotransferase (AST) >40 U/Liter. Immunosuppression was defined as AIDS, history of organ transplant, neutropenia, known malignancy, taking immunosuppressive medication and congenital immunodeficiency. Pregnancy was considered an immunosuppressed state.

A modified Acute Physiologic and Chronic Health Evaluation (APACHE 2) score was calculated using age, temperature, mean arterial blood pressure, respiratory rate, potassium, creatinine, acute renal failure, and comorbid conditions to estimate severity of illness [16]. PaO2 was estimated using pulse oximetry oxygen saturation results and hematocrit was calculated by multiplying the hemoglobin times three.

### Statistical analysis

All statistical analyses were performed using Stata software (Version 13.1, College Station, TX). The percent distribution of clinical variables among patients who survived and those who died were compared using the Fisher exact test. A multivariate logistic regression was done on treatments administered and severity of illness to determine which treatments were associated with survival. Mycophenolate mofetil was not included in this logistic regression analysis because 100 % of patients receiving mycophenolate mofetil survived. The association between severity of illness and treatments administered was assessed by performing a linear regression of treatments administered onto the modified APACHE 2 score.

## Results

### Demographic and exposure characteristics

There were a total of 51 cases, thirty patients (58.8 %) of whom were Saudi nationals, and 21 (41.2 %) were foreign nationals. The median age was 54 years old (IQR 36.5–58). Most were male (*n* = 40, 78.4 %). Twenty-one patients (41.2 %) had exposure to a known patient with MERS coronavirus and 12 (23.5 %) were healthcare workers. None of the patients had animal exposure. Two patients (3.9 %) were on pilgrimage to Mecca.

Overall, 71 % of patient had at least one co-morbid condition. Seventeen patients had diabetes (33.3 %), 25 had hypertension (49 %), 14 (27.5 %) had end stage renal disease, eight (15.7 %) had coronary artery disease and six (11.8 %) patients were immunosuppressed, two of whom were pregnant.

### Clinical and laboratory findings

Forty-nine patients (96 %) had documented fever, 41 (80.4 %) had cough, and 46 (90 %) reported shortness of breath. Thirteen patients (25.5 %) had diarrhea, 12 (23.5 %) had vomiting and 5 (9.8 %) complained of sore throat. The clinical findings on presentation included eight patients (15.7 %) with hypotension, 39 (76.9 %) with tachypnea and 17 patients (33 %) with hypoxia. As previously reported, laboratory findings were non-specific. Fourteen patients (27.5 %) were anemic, 15 (29.4 %) were thrombocytopenic, and 28 (54.9 %) were leukopenic. Many patients had liver function abnormalities. Twenty-three (45.1 %) had elevated ALT and 35 (68.6 %) had an elevated AST. Thirty-two patients (62.8 %) had an elevated LDH, 24 patients (47.1 %) had an elevated CK and 21 patients (41.2 %) had an elevated creatinine.

### Treatments administered

Patients received a variety of novel treatments including immunosuppressants and antivirals. Forty-two (82.4 %) patients received broad-spectrum antibiotics and five (9.8 %) received hydrocortisone. Thirty one patients received antiviral treatment. Twenty-three patients (45.1 %) were treated with interferon beta, eight (15.7 %) were treated with interferon alpha. A variety of anti-viral combinations were used. Eight patients (15.7 %) received mycophenolate mofetil, seven of these patients received it in combination with interferon beta. Nineteen (37.3 %) patients required intensive care unit (ICU) care, and 10 patients received extracorporeal membrane oxygenation (ECMO). All patients treated in the ICU and all patients receiving ECMO died.

### Univariable association of demographic, clinical features and treatment with death

In this recent cohort, when comparing survivors to non-survivors, survival was associated with male gender, vomiting on admission, elevated respiratory rate, abnormal lung exam on physical exam, working as a healthcare worker, history of hypertension, elevated ALT, clearance of MERS CoV on repeat PCR testing, and receiving mycophenolate mofetil or beta interferon (Table 1). In contrast, markers of severe disease like hypotension, hypoxemia, chest radiographic abnormalities, leukocytosis, elevated creatinine, thrombocytopenia, anemia, renal failure were associated with death.

**Table 1:** Predictors for poor outcome among 51 MERS CoV cases in KSA 2014

	Total	Survival	Death	*P* value
Demographics and Epidemiology				
Age < = 30	7 (13.7 %)	6 (85.7 %)	1 (14.29 %)	0.187
Age 31-60	32 (62.8 %)	22 (64.7 %)	12 (35.3 %)	
Age >60	10 (19.6 %)	4 (40 %)	6 (60 %)	
Gender - male	40 (78.4 %)	22(55 %)	18(45 %)	0.037
Known MERS exposure	21 (41.2 %)	12 (57.1 %)	9 (42.9 %)	0.563
Healthcare worker	12 (23.5 %)	11 (91.7 %)	1 (8.3 %)	0.020
Umrah patient	2 (3.9 %)	2 (100 %)	0 (0 %)	0.523
Co-morbid conditions				
Diabetes	17 (33.3 %)	9 (52.9 %)	8 (47.1 %)	0.365
Hypertension	25 (49 %)	10 (40 %)	15 (60 %)	0.001
End stage renal disease	14 (27.5 %)	6 (42.9 %)	8 (57.1 %)	0.106
Coronary artery disease	8 (15.7 %)	3 (37.5 %)	5 (62.5 %)	0.131
Immunosuppression	6 (11.8 %)	5 (83.3 %)	1 (16.7 %)	0.392
Signs and Symptoms				
Runny nose	6 (11.8 %)	5 (83.3 %)	1 (16.67 %)	0.392
Cough	41(80.4 %)	26 (63.4 %)	15(36.6 %)	1.000
Diarrhea	13 (25.5 %)	11 (84.6 %)	2 (15.4 %)	0.096
Vomiting	12 (23.5 %)	11 (91.7 %)	1 (8.3 %)	0.020
Sore throat	5 (9.8 %)	3 (60 %)	2 (40 %)	1.000
Abnormal lung exam	20 (39.2 %)	17 (85 %)	3 (15 %)	0.016
Subjective fever	49 (96.1 %)	30 (61.2 %)	19 (38.8 %)	0.523
Temperature >38 Celsius	25 (49 %)	13 (52 %)	12 (48 %)	0.153
Respiratory rate >16	39 (76.5 %)	21 (53.9 %)	18 (46.2 %)	0.020
Blood pressure < 90/60 mm Hg	8 (15.7 %)	0 (0 %)	8 (100 %)	0.000
Oxygen saturation < 90 %	17 (33.3 %)	4 (23.5 %)	13 (76.5 %)	0.000
Laboratory findings				
Hemoglobin < 10 g/dL	14 (27.5 %)	4 (28.6 %)	10 (71.4 %)	0.003
White Blood cell count < 5,000 cells/mm^3^	28 (54.9 %)	21 (75 %)	7 (25 %)	0.080
White Blood cell count > 10,000 cells/mm^3^	9 (17.6 %)	2 (22.2 %)	7 (77.8 %)	0.009
Platelets < 150,000/mm3	15 (29.4 %)	9 (60 %)	6 (40 %)	1.000
ALT > 50 U/L	23 (45.1 %)	14 (61 %)	9 (39.1 %)	1.000
AST > 40 U/L	35 (68.6 %)	18 (51.4 %)	17 (48.6 %)	0.015
LDH > 300 U/L	32 (62.7 %)	18 (56.3 %)	14 (43.8 %)	0.247
Creatinine > 1.5 mg/dL	21 (41.2 %)	8 (38.1 %)	13 (61.9 %)	0.003
CK > 200 U/L	24 (47.1 %)	13 (54.2 %)	11 (45.8 %)	0.261
Potassium > 4.5 mmol/L	17 (33.3 %)	8 (47.1 %)	9 (52.9 %)	0.131
Negative repeat PCR swab	31 (60.8 %)	30 (96.8 %)	1 (3.23 %)	0.000
Radiology				
CXR – Right upper lobe infiltrate	17 (33.3 %)	5 (29.4 %)	12 (70.6 %)	0.001
CXR – Right lower lobe infiltrate	32 (62.7 %)	13 (40.6 %)	19 (59.4 %)	0.000
CXR – Left upper lobe infiltrate	19 (37.3 %)	7 (36.8 %)	12 (63.2 %)	0.006
CXR – Left lower lobe infiltrate	31 (60.8 %)	13 (41.9 %)	18 (58.1 %)	0.000
Treatments administered				
Interferon beta	23 (45.1 %)	18 (78.3 %)	5 (21.7 %)	0.047
Interferon alpha	8 (15.7 %)	6 (75 %)	2 (25 %)	0.694
Any interferon	31 (60.8 %)	24 (77.4 %)	7 (22.6 %)	0.009
Ribavirin	19 (37.5 %)	13 (68.4 %)	6 (13.6 %)	0.564
Antibiotics	42 (82.4 %)	26 (61.9 %)	16 (38.1 %)	1.000
Hydrocortisone	5 (9.8 %)	2 (40 %)	3 (60 %)	0.348
Mycophenolate mofetil	8 (15.7 %)	8 (100 %)	0 (0 %)	0.019
ICU stay during admission	19 (37.3 %)	0 (0 %)	19 (100 %)	0.000
Extracorporeal Membrane Oxygenation	10 (19.6 %)	0 (0 %)	10 (100 %)	0.000

### Treatments given

Treatments given were based as indicated based on the clinical assessment of the infectious disease consult team. Thirty-one patients received antivirals, ribavirin or alpha or beta interferon, and 13 patients received immunosuppressive medication. Most patients received a combination of alpha interferon and ribavirin (5, 9.8 %), beta interferon and ribavirin (10, 19.5 %) or beta interferon alone (11, 21.6 %). Two patients received alpha interferon alone (3.9 %). Eight patients received mycophenolate mofetil (15.7 %) and seven of them received this in combination with beta-interferon. Five patients received hydrocortisone; two in combination with beta interferon and ribavirin and 3 in combination with alpha interferon and ribavirin. All eight patients given mycophenolate mofetil survived therefore mycophenolate mofetil could not be evaluated in this model.

### Impact of treatments

While the results of the univariable analysis demonstrated improved survival in patients treated with beta-interferon and mycophenolate mofetil, the multivariable analysis which included a marker of severity of illness, demonstrated a strong association between severity of illness and reduced survival, and no association between treatment with beta interferon and survival. Mycophenolate mofetil was not evaluable in this model (Table 2). In analyzing the relationship between severity of illness and treatments administered, beta interferon and mycophenolate mofetil were given to less severely ill patients (Table 3)

**Table 2:** Multivariable analysis of treatments and their impact on mortality

	Odds ratio	Confidence interval	*P* value
Beta interferon	0.68	0.04–10.28	0.778
Alpha interferon	0.47	0.02–10.38	0.630
Hydrocortisone	2.92	0.13–63.62	0.495
Ribavirin	0.66	0.04–12.36	0.779
Modified APACHE 2 score	1.60	1.18–2.17	0.002

**Table 3:** Univariable analysis of the impact of severity of illness on treatments administered

	Risk association	Confidence interval	*P* value
Beta interferon	– 4.62	−8.40, −0.84	0.018
Alpha interferon	– 1.24	−6.71, 4.24	0.652
Ribavirin	0.78	−3.34,4.90	0.704
Viral treatment	– 5.98	−9.73, −2.23	0.002
Mycophenolate mofetil	– 7.91	−12.90, −2.91	0.003
Hydrocortisone	3.03	−3.62,9.68	0.364

## Discussion

MERS-CoV is an emerging disease for which the initial epidemiology has been described, but in-depth clinical studies and the role of therapy in incompletely understood. While the clinical features for MERS-CoV have been described in several large case series [6–14], there is a paucity of literature on therapy. Our results from a relatively large number of patients demonstrate similar clinical features and mortality to previous studies [6–14]. In our cohort, treatment with beta interferon and mycophenolate mofetil may be predictive of survival, but the greatest predictor of survival is the severity of illness on presentation.

Improved diagnostics have demonstrated an expanded spectrum of disease that includes less severe cases than previously reported. We now understand that MERS-CoV causes an acute respiratory disease syndrome and one third of patients present with gastrointestinal symptoms [14]. Fever has been seen in 62–87 % of patients, cough in 55–87 %, and gastrointestinal symptoms in 26–35 %. Seventy-six to 96 % percent of patients have had comorbid illnesses, most commonly chronic renal failure, diabetes and heart disease [7–15]. This may be partially related to the epidemiology of increased disease transmission in healthcare settings rather than a true host risk factor. Laboratory findings have been non-specific and consistent with other viral infections. Thrombocytopenia (75 %) and lymphopenia (58 %) have been commonly described in these patients [7, 9–13, 15]. Forty three percent had acute kidney injury [7, 11–13, 17] and 76–100 % had CXR abnormalities with bibasilar infiltrates as the most common finding [8–13, 15, 18]. The outcomes in these more severely ill patients remain poor. Between 50–90 % required ICU care [10, 11, 13, 15] and 67–100 % in the ICU setting required invasive ventilation for a median of 7–16 days [8, 10, 12]. In addition to mechanical ventilation, several patients have received extracorpeal membrane oxygenation (ECMO) to support ventilation. From non-randomized data from the World Health Organization, five out of six patients receiving ECMO died [9]. Fifty-eight to 75 % required renal replacement therapy [11, 12, 17] and 30–60 % of hospitalized patients died [7–15]. The severity of illness can be partially explained by the widespread lung disease caused by MERS-CoV and it appears that mortality in those patients requiring intensive care is extremely high. Although no autopsy data is available, in explanted lung, infection with MERS-CoV causes widespread infection and alveolar disease [19, 20].

The clinical features in our cohort similarly also show a high proportion of patients with fever (96 %) and cough (80.4 %) shortness of breath (90 %), and almost one third of patients (29.4 %) with gastrointestinal symptoms. Our cohort consisted of ill patients with hypotension (15.7 %), tachypnea (76.9 %) and hypoxia (33 %). Thirty seven percent required ICU care and 10 patients received ECMO. Similar to previous results, all of the patients who received ECMO died [9].

There is no known effective treatment for MERS CoV. Many compounds have been screened in vitro for possible activity against this coronavirus [21–24], however, the in vivo efficacy has not been subjected to clinical investigation.

In vitro data suggests that MERS-CoV inhibits host interferon production through various molecular pathways [25–30] mycophenic acid, the active agent of prodrug mycophenolate mofetil, and cyclosporine strongly inhibit MERS coronavirus in human and monkey cell lines even more so than they inhibit SARS coronavirus [24, 31–33]. Interferon alpha and interferon beta reduce MERS coronavirus replication in explanted lung tissue [19]. In vivo, comparing host response in two patients with MERS coronavirus and differing outcomes, the patient who was able to clear MERS CoV infection was able to mount an interferon response and the patient who died had low levels of interferon alpha suggesting a therapeutic role for interferon [34]. The combination of interferon alpha and ribavirin has been used successfully in rhesus monkeys infected with MERS coronavirus [35], and in a few small case series [36–38]. Beta-interferon seems to be an even more potent inhibitor of MERS coronavirus in vitro [19] [24, 31–33]. One small study with exceptionally high mortality rates using interferon beta for treatment found no difference in mortality between interferon beta use and interferon alpha use [39]. Our data, albeit from a retrospective cohort support the findings that interferon beta is associated with a decrease in mortality.

There are limited data on the efficacy of treatment regiments for this virulent disease. We present data from a retrospective cohort of ill patients with Mers-CoV and the results of the evaluation of the clinical efficacy of beta interferon beta, alpha interferon, ribavirin and mycophenolate mofetil in addition to routine supportive care. Forty five percent of patents (23 patients) received interferon beta and in this cohort, sixteen percent of patients received interferon alpha (8 patients) and 37 % of patients (9 patients) received ribavirin, either in conjunction with interferon alpha or interferon beta, and 8 patient received mycophenolate mofetil. Patients receiving beta interferon and mofetil had improved survival, however this was confounded by the severity of illness on presentation for beta interferon. All of the patients who received mycophenolate mofetil survived however because of the small number, we could not analyze the independent efficacy of mycophenolate mofetil.

While this is a relatively large series of MERS-CoV cases, the primary limitation of our study is that it is a retrospective review of cases and not a randomized trial and thus subject to confounding as seen in our cohort. We used a modified APACHE 2 score without all of the clinical variables, which may have underestimated the association of severity of illness with reduced survival. Importantly, the mortality in patients receiving additional therapies that modulate the immune response was low. All of the eight patients who received mycophenolate mofetil in our study survived. Hence, it may be reasonable to further study this agent in controlled trials.

## Conclusions

This observational study investigates novel treatment options like beta interferon and mycophenolate mofetil for MERS-CoV in humans which have in vitro activity. Our cohort demonstrated severity of illness is an important effect modifier and needs to be considered in evaluating novel agents. To better assess the efficacy of these therapies, international prospective randomized trials with adequate numbers of patients are needed to further evaluate the impact of these treatments in addition to routine supportive care when compared to other treatment options.

### Ethics approval

This study was reviewed and approved by Johns Hopkins University Institutional Review Board and the Directorate of Health Affairs.

### Availability of data and materials

Data supporting the findings are in the manuscript, additional data available upon request.

## Abbreviations

AIDS: acquired immune deficiency syndrome
ALT: alanine transaminase
APACHE 2: acute physiologic and chronic health evaluation
AST: aspartate aminotransferase
CXR: chest x ray
ECMO: extracorporeal membrane oxygenation
ICU: intensive care unit
LDH: lactate dehydrogenase
MERS Co-V: Middle Eastern Respiratory Syndrome coronavirus
PCR: polymerase chain reaction
